# Antifungal Activity of Lactic Acid Bacteria Combinations in Dairy Mimicking Models and Their Potential as Bioprotective Cultures in Pilot Scale Applications

**DOI:** 10.3389/fmicb.2018.01787

**Published:** 2018-08-07

**Authors:** Marcia Leyva Salas, Anne Thierry, Mathilde Lemaître, Gilles Garric, Marielle Harel-Oger, Manon Chatel, Sébastien Lê, Jérôme Mounier, Florence Valence, Emmanuel Coton

**Affiliations:** ^1^Université de Brest, EA 3882 Laboratoire Universitaire de Biodiversité et Ecologie Microbienne, ESIAB, Technopôle Brest-Iroise, Plouzané, France; ^2^UMR1253 Science et Technologie du Lait et de l'Œuf, INRA, Agrocampus Ouest, Rennes, France; ^3^Applied Mathematics Department, Agrocampus Ouest, Rennes, France

**Keywords:** biopreservation, dairy products, propionibacteria, *Lactobacillus harbinensis*, *L. plantarum*, *L. rhamnosus*

## Abstract

Consumer's demand for naturally preserved food products is growing and the use of bioprotective cultures is an alternative to chemical preservatives or a complementary tool to hurdle technologies to avoid or delay fungal spoilage of dairy products. To develop antifungal cultures for the dairy product biopreservation, experiments were conducted both *in vitro* and *in situ*. Firstly, the antifungal activity of 32 strains of lactic acid bacteria (LAB) and propionibacteria was screened alone, and then on combinations based on 5 selected lactobacilli strains. This screening was performed in yogurt and cheese models against four major spoilage fungi previously isolated from contaminated dairy products (*Penicillium commune, Mucor racemosus, Galactomyces geotrichum*, and *Yarrowia lipolytica*). Selected combinations were then tested as adjunct cultures in sour cream and semi-hard cheeses produced at a pilot scale to evaluate their antifungal activity during challenge tests against selected fungal targets (*P. commune, M. racemosus*, and *Rhodotorula mucilaginosa*) and shelf life tests; and their impact on product organoleptic properties. The screening step allowed selecting two binary combinations, A1 and A3 composed of *Lactobacillus plantarum* L244 and either *Lactobacillus harbinensis* L172 or *Lactobacillus rhamnosus* CIRM-BIA1113, respectively. *In situ* assays showed that the A1 combination delayed the growth of *P. commune, M. racemosus* and *R. mucilaginosa* for 2–24 days on sour cream depending of the antifungal culture inoculum, without effect on organoleptic properties at low inoculum (10^6^ colony-forming units (CFU)/mL). Moreover, the A1 and A3 combinations also delayed the growth of *P. commune* in semi-hard cheese for 1–6 days and 1 day, respectively. Antifungal cultures neither impacted the growth of starter cultures in both sour cream and cheese nor the products' pH, although post acidification was observed in sour cream supplemented with these combinations at the highest concentrations (2.10^7^ CFU/mL). The combination of both *in vitro* and *in situ* screening assays allowed developing 2 antifungal combinations exhibiting significant antifungal activity and providing future prospects for use as bioprotective cultures in dairy products.

## Introduction

Food spoilage by fungi (molds or yeasts) is responsible for considerable food waste and economical losses. Among food products, fermented dairy products can be affected by fungal spoilers resistant to low pH and able to grow at low storage temperatures (<10°C). These contaminants exhibit proteolytic and lipolytic capacities, and consume the dairy product main sugars and organic acids (lactic and citric acids), thus impacting the product organoleptic qualities (Pitt and Hocking, 2009). Fungal spoilers can affect the product aspect (presence of thallus or yeast colonies, gas production), texture, odor, and flavor (Ledenbach and Marshall, 2009; Pitt and Hocking, 2009). In this context, the main fungal genera encountered in contaminated dairy products and production environments correspond to *Penicillium, Mucor*, and *Cladosporium* for molds, and *Candida, Meyerozyma*, and *Yarrowia* for yeasts (Pitt and Hocking, 2009; Garnier et al., 2016).

Preclusion of fungal spoilage in food currently relies on prevention methods (e.g., implementation of Hazard Analysis Critical Point -HACCP-), hurdle technologies (e.g., heat treatments, water removal, modified atmosphere packaging, salting, fermentation) and the use of chemical preservatives, including benzoate, propionate, sorbate, nitrate, and sulfites (Silva and Lidon, 2016). For fungal control in dairy products, potassium sorbate and natamycin (E235), a microbial food preservative produced by *Streptomyces natalensis* belonging to the group of polyene macrolide antimycotics, are the main preservatives used. Natamycin is approved by most countries for use as a cheese surface treatment while its addition into other foods and the permitted applications differ per country/region (Stark and Tan, 2003). Agri-food industries rely mainly on chemical preservatives to control microbial contaminant growth and extend the product shelf life, but a strong societal demand for less processed and preservative-free food has emerged, and additive regulations are constantly evolving to limit their use (Fuselli et al., 2012). The latter aspects, combined with the necessity to produce food products complying with high safety and quality standards, have led to the search for alternatives to the use of chemical preservatives. Among the explored natural alternatives, biopreservation, corresponding to use of microorganisms and/or their antimicrobial metabolites (Stiles, 1996), has recently attract much interest.

In the dairy product context, lactic acid bacteria (LAB) and propionibacteria (PAB), which are naturally present or used as cultures in these matrices, play a key role in the fermentation process. Fermentation is a “natural” preservation technique that improves food safety, nutritional value and specific organoleptic features (e.g., eye formation in Swiss-type cheeses by propionibacteria) (Bourdichon et al., 2012; Fröhlich-Wyder et al., 2017). Bacterial fermentation induces acidification, due to the production of organic acids (e.g., lactic, acetic, or propionic acids), which play a role in fermented food biopreservation (Batish et al., 1997; Leroy and De Vuyst, 2004). These organic acids as well as other metabolites such as phenyllactic, hydroxyphenyllactic, benzoic acids, fatty acids, volatile compounds (e.g., diacetyl, acetoin), cyclic dipeptides, hydrogen peroxide, reuterin, and/or proteinaceous compounds have exhibited an antifungal activity (Crowley et al., 2013; Leyva Salas et al., 2017). The use of LAB and PAB to reduce levels or fully replace antifungal chemical preservatives is clearly an alternative of interest as these microorganisms are recognized as safe (as confirmed by their Qualified Presumption of Safety -QPS- or Generally Recognize As Safe -GRAS- status), are known to have a long history of safe use and obviously exhibit antifungal properties (Bourdichon et al., 2012). In this context, several studies of LAB and PAB as bioprotective cultures with antifungal activity have been reported during the last years in various dairy products (yogurt, semi-hard ewe milk cheese, cottage cheese, and cheddar cheese; Delavenne et al., 2013, 2015; Li et al., 2013; Cheong et al., 2014; Lynch et al., 2014; Aunsbjerg et al., 2015; Gómez-Torres et al., 2016; Fernandez et al., 2017) Noteworthy, despite the number of reported studies, only a few number of antifungal cultures are available on the market due to several constraints before commercialization of a strain of interest (e.g., gap between *in vitro* and *in situ* activity, undesirable organoleptic impacts on the products, safety evaluation and cell viability) (Leyva Salas et al., 2017).

The aim of this study was to develop bioprotective cultures with antifungal activity suitable for use in the dairy industry as adjunct cultures. To do so, a scale-up approach, going from antifungal activity screening in dairy models to pilot scale applications in actual dairy products, was performed. Several aspects were considered in order to get close to actual dairy industry conditions: (i) antifungal activity screening of 32 strains compatible with a use as adjunct cultures, (ii) use of two dairy models for the *in vitro* tests, (iii) antifungal activity test against fungal targets isolated from contaminated dairy products, (iv) assessment of the antifungal activity of bioprotective cultures at a pilot scale in two dairy products, and (v) evaluation of the impact of the antifungal bioprotective cultures on the starter cultures as well as the pH and organoleptic traits of the final products.

## Materials and methods

### Microorganisms and culture conditions

LAB (23 strains) and PAB (9 strains) (Table 1) were obtained from the Université de Bretagne Occidentale (UBOCC, France) and CIRM-BIA (INRA, France) culture collections. They all corresponded to strains that were previously shown to exhibit antifungal activity, either directly (Delavenne et al., 2012) or indirectly (use of fermentates, i.e., ingredients produced by the fermentation of a dairy matrices) (Garnier et al., 2018). These strains, presenting diversity at the species (19 *Lactobacillus*, 4 *Leuconostoc*, and 9 *Propionibacterium*) and intraspecific level, were selected based on their compatibility with dairy technology. Moreover, three strains isolated on de Man-Rogosa-Sharpe (MRS) agar from commercial bioprotective cultures were used as reference antifungal strains in yogurt, cheese model and sour cream. They corresponded to *Lactobacillus rhamnosus* CIRM-BIA1759 and *Lactobacillus paracasei* CIRM-BIA1761 isolated from the commercial culture CC1, while *Lactobacillus plantarum* CIRM-BIA1758 was isolated from the commercial culture CC2. Culture X1 corresponded to a 1:1 combination of the *L. paracasei* CIRM-BIA1759 and *L. rhamnosus* CIRM-BIA1761 strains, while X2 corresponded to *L. plantarum* CIRM-BIA1758 alone. For semi-hard cheese production, a commercial culture CC3 (consisting of the same species and purchased from the same supplier that CC1) was directly applied as recommended by the supplier. All strains were stored at −80°C in MRS broth (BD Difco laboratories, Sparks, MD, USA) supplemented with glycerol (20%) (Thermo Fischer Scientific, Waltham, MA, USA). Before inoculation as adjunct cultures, strains were cultivated twice in MRS broth for LAB and in yeast extract lactate (YEL) medium for PAB at 30°C for 24 and 48 h, respectively. Culture concentration was then adjusted to the target concentrations with sodium chloride peptone broth (Merck, Darmstadt, Germany). After centrifugation, the cells were resuspended in sterile skimmed milk. Various LAB commercial starters (i.e., used for their technological properties) were used for screening tests and pilot scale experiments. They corresponded to MA016 (*Lactococcus lactis* subsp. *cremoris* and *L. lactis* subsp. *lactis*, Elimeca, Thoissey, France); MM100 (*L. lactis* subsp. *lactis, L. lactis* subsp. *cremoris*, and *L. lactis* subsp. *lactis* biovar *diacetylactis*, Choozit, Danisco, Sassenage, France); MD88 (*L. lactis* subsp. *lactis* biovar *diacetylactis*, Choozit, Danisco, Sassenage, France); MY800 (*Streptococcus thermophilus, Lactobacillus delbrueckii* subsp. *lactis* and *L. delbrueckii* subsp. *bulgaricus*, Choozit, Danisco, Sassenage, France) and PAL YOG 1-30D (*S. thermophilus* and *Lactobacillus delbrueckii* subsp. *bulgaricus*, Standa, Caen, France).

**Table 1:**
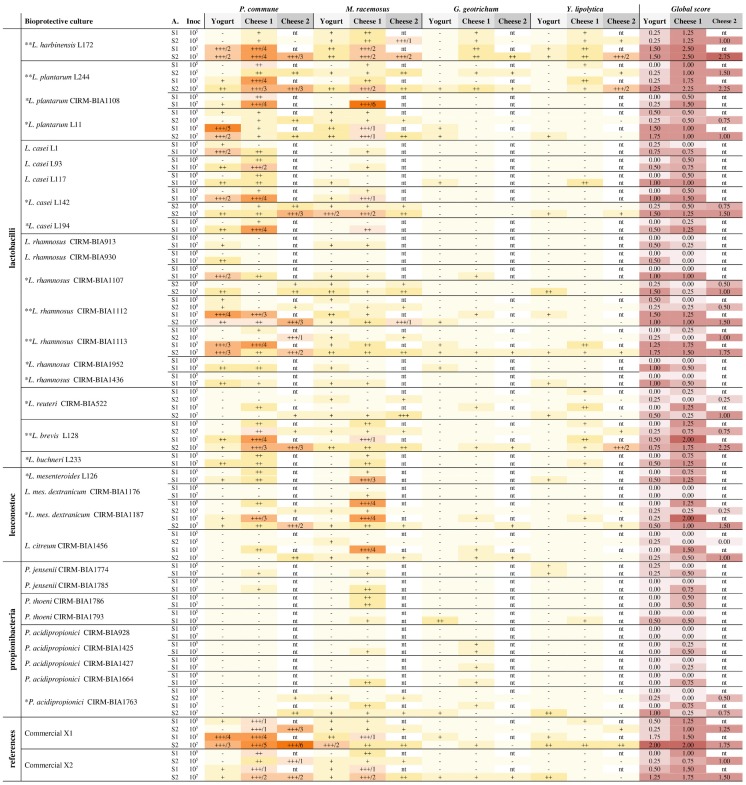
Antifungal activity of 32 strains and commercial reference cultures against 4 fungal targets in yogurt and cheese model.

*L., Lactobacillus or Leuconostoc; P., Propionibacterium. X1 and X2, antifungal reference cultures (for details see the Materials and Methods section)*.

*Antifungal activity from the different assays (A): S1, first screening of 32 strains; S2, Second screening of 12 preselected strains. Inoc, inoculum CFU/mL*.

^*^ Strains with high global score; ^**^ 5 selected strains for combinations.

Antifungal activity in the dairy products: Yogurt, Cheese 1 and 2 with commercial starters MY800, MA16 and MD88, respectively.

Nt, not tested.

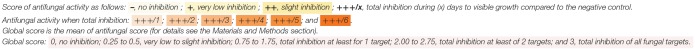

The 10 fungal targets used, 4 filamentous molds and 6 yeasts, were from the UBOCC collection (Plouzané, France). They corresponded to *Candida parapsilosis* UBOCC-A-216002, *Galactomyces geotrichum* UBOCC-A-216001, *Meyerozyma guilliermondii* UBOCC-A-216003, *Mucor racemosus* UBOCC-A-116002, *Penicillium commune* UBOCC-A-116003, *Penicillium bialowiezense* UBOCC-A-117365, *Didymella pinodella* UBOCC-A-116004, *Yarrowia lipolytica* UBOCC-A-216006, *Rhodotorula mucilaginosa* UBOCC-A-216004 and *Trichosporon asahii* UBOCC-A-216005. Filamentous fungi were cultivated and then stored at −80°C as a spore or cell suspension in sterile glycerol as previously described (Delavenne et al., 2012), and yeasts were pre-cultivated in potato dextrose broth (PDB, Difco, Becton Dickinson, Sparks, MD, USA). Spore or cell concentrations in suspension were determined using a haemocytometer (Malassez, Preciss, Paris, France) and adjusted with sodium chloride peptone broth to 5.10^3^ spores or cells/mL.

### Antifungal activity screening of bacterial strains

**Figure 1:**
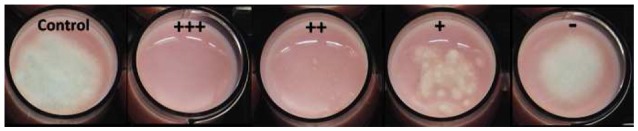
Example of the antifungal inhibition scoring in yogurt against *P. commune*. Control, yogurt with no antifungal culture; +++, total inhibition; ++, slight inhibition; +, very low inhibition; –, no inhibition.

Antifungal activity was screened using the high-throughput method developed by Garnier et al. (2018) on two dairy matrices: the same cheese-mimicking matrix used by the latter authors and a yogurt. The cheese-mimicking matrix (hereafter named model cheese) was prepared from a heat-treated salted 0.7% (w/w) ultrafiltered milk retentate, kept at −20°C until use. The model cheese was defrosted at 48°C for 3 min, then 10 mL/L of a pH indicator (sterile solution of Litmus 50 g/L), 10^6^ colony forming unit (CFU)/mL of commercial starters (either MA016, MM100 or MD88) and 1.5 mL/L of 5X diluted and filtered (0.22 μm) chymosin rennet (Maxiren 180, DSM Food Specialties, The Netherlands) were added to the thawed model cheese. After vigorous homogenization for 1 min, it was distributed into 24-well plates (2 mL/well), cell suspensions in milk of the tested strains were added or not (positive and negative fungal growth control wells) at 10% v/v at a final concentration of 10^5^ and 10^7^ CFU/mL (2 wells per concentration and 1 strain by column) and plates were incubated for 1 h at 30°C then for 3 days at 20°C with a final pH of 4.8 ± 0.1. The screening method was also adapted to yogurt. To do so, yogurt was prepared from semi-skimmed milk (Candia, France) supplemented with skimmed milk powder at 4% (Carrefour, France). After heat treatment for 30 min at 85°C, milk was rapidly cooled to 45°C before adding 0.1 g/L of the lyophilized starter culture (either MY800 or PAL YOG 1-30D) and pH indicator. After homogenization, the preparation was distributed into 24-well plates (2 mL/well) and the antifungal strain suspensions were added under the same conditions as for the model cheese. Plates were then incubated at 42°C during 4 or 6 h (for starters MY800 and PAL-YOG-1-30D, respectively) until pH 4.97 ± 0.03 was reached. For the two dairy models, at the end of fermentation, exudate was removed and 50 spores/cells of either *M. racemosus, P. commune, Y. lipolytica*, or *G. geotrichum* were spotted at the center of each well (1 fungi tested/plate) except for the positive control wells. Antifungal activity was assessed by visually evaluating fungal growth in comparison with the negative control, after incubation for up to 8 days at 12 and 10°C (for cheese and yogurt plates, respectively). Antifungal activity scoring was done using a qualitative system presented in Figure 1 and by recording the days of total inhibition until fungal growth was visible. Moreover, a global score was calculated for each tested culture at a given inoculum. This global score corresponded to the sum of a numeric equivalent of the antifungal score (0, equivalent to –, corresponded to no inhibition and 3, equivalent to +++, corresponded to total inhibition) divided by the total number of tested fungal targets.

A first antifungal activity screening was performed for the 32 selected strains in the two dairy models with only one commercial starter per model (MY800 and MA016 for yogurt and cheese, respectively). In a second step, the impact of the commercial starters on the observed antifungal activity of 12 strains was evaluated using 3 other commercial starters. Then, 5 strains of interest were selected to be evaluated in combinations.

### Evaluation of the antifungal activity of strain combinations

Ten binary and three ternary combinations, based on a selection of 5 strains, were tested using the same screening method on yogurt and model cheese with only one starter for each model, and compared to the individual strains to evaluate combination effects. Combinations of interest were then tested for antifungal activity at four inoculum levels ranging from 10^6^ to 2.10^7^ CFU/mL in the two models in the presence of different commercial starters. Then, to evaluate the action spectrum of the selected antifungal combinations and pure cultures, six supplementary fungal targets, namely *D. pinodella, C. parapsilosis, M. guilliermondii, P. bialowiezense, R. mucilaginosa*, and *T. asahii*, were studied in yogurt and cheese models.

### Antifungal activity in pilot scale conditions

Antifungal activity of the selected combinations was then determined in two types of dairy products, sour cream and semi-hard cheese, produced at a pilot scale at the Dairy Platform of INRA Rennes, France.

#### Challenge-tests in sour cream

Sour cream (final fat 35%) was produced from 25 L of pasteurized (20 s at 75°C), skimmed and standardized cow milk (50°C, 35% fat). Skimmed milk was then homogenized (65°C, 50 bars, and then 5 bars) and inoculated with a commercial starter (MM100) at 3 U/100 L, the selected antifungal combinations (either 10^6^, 5.10^6^ or 2.10^7^ CFU/mL) or the X1 combination (10^6^ CFU/mL) before packaging in 100 g plastic tubs with mobile lids. After overnight incubation (22 h at 23°C), sour cream samples were challenged. For positive controls, instead of the bioprotective cultures, potassium sorbate (Sigma-Aldrich, Saint-Louis, MO, USA) was added at 0.08% after acidification and mixed using a sterile spatula. Negative control corresponded to the sour cream containing only the technological starter. For challenge-tests, 50 fungal spores of either *P. commune* UBOCC-A-116003 or *M. racemosus* UBOCC-A-116002 were inoculated on the sour cream surface and incubated at 10°C. Fungal growth was then visually evaluated daily during 4 weeks. For a third fungal target, *R. mucilaginosa* UBOCC-A-216004, cream tubs were inoculated at 2 CFU/g and mixed with a sterile spatula. Yeast growth was evaluated by enumeration on YEGC medium, containing 20 g/L of glucose (Sigma-Aldrich, Saint-Louis, MO, USA), 5 g/L of yeast extract (Merck, Darmstadt, Germany) and 0.5 g/L of chloramphenicol (Sigma-Aldrich, Saint-Louis, MO, USA) after incubation for 7 days at 10°C. A number of non-challenged cream tubs were stored at 4°C for bacterial control, pH control, shelf life test and sensory analyses.

#### Challenge-tests in semi-hard cheese

Two replicate cheese trials were undertaken on two separate weeks. For each trial, four vats of cheese were produced, one for each antifungal combination and one control cheese without antifungal culture. Cheese were manufactured as follows. Cow milk was pasteurized at 72°C for 20 s, standardized to 30 g fat/kg milk, added with 36 mg Ca per kg (20 mL per 100 kg milk of a 500 g /L CaCl_2_ solution), then cooled to 4°C and stored overnight. Prior to cheese making, the milk was pumped into 30 L cylindrical jacketed stainless steel cheese vats, with variable speed cutting and stirring (Frominox, Assat, France) and warmed to 33°C. The MA016 commercial starter, previously suspended in UHT semi-skimmed milk and let to rehydrate for 1 h at 20°C, was added to the cheese milk at 0.5 U/100 kg (i.e., ~10^6^ CFU/mL). Antifungal cultures were added at 5.10^6^ CFU/mL (each strain at 2.5.10^6^ CFU/mL for the A1 and A3 combinations, and the commercial culture CC3). After around 30 min pre-ripening at 33°C (pH 6.57–6.59), 0.25 mL/kg of animal rennet (520 mg/L chymosin, Carlina 145/80, Dupont Danisco, Dangé, Saint Romain, France) diluted in deionized water was added. After coagulation (34–37 min), curd was cut to the size of corn grains, washed with warm water, cooked for 20 min at 35°C, and drained. Curd was pre-pressed at 60 kPa for 30 min, molded (700 g curd per mold), and pressed at 300 kPa for 30 min followed by 600 kPa for 90 min. The obtained cheeses remained in the mold overnight, the temperature decreased from ~25 to ~20°C. At demolding, cheeses had a pH of 5.11 ± 0.04 and contained 51.41 ± 1.13% dry matter (pH of 5.14 ± 0.03 and 5.09 ± 0.04 and dry matter of 50.79 ± 0.75, and 52.11 ± 1.02, for cheese trials 1 and 2, respectively) and 45% fat in dry matter. Cheeses were salted by immersion at 20°C for 4 h in a sterile brine containing 33% (m/m) NaCl, 36 mg/L Ca, pH 5.2, and were let to dry overnight at room temperature (20–25°C) under a laminar flow hood. The external part of cheeses (3 mm thickness) was then sterilely discarded and their surface smeared with a smear preparation containing the commercial cultures *Debaryomyces hansenii* CHOOZIT DH, *Kluyveromyces lactis* CHOOZIT KL71, and *Brevibacterium linens* CHOOZIT SR3, (Danisco, Dangé Saint-Romain, France) at 0.01% w/w each in a sterile 0.1% NaCl solution, pH 5.2. Cheeses were then dried for 1 h at room temperature under laminar flow hood and then transferred to ripening chambers (12°C). After 1 day in the ripening chambers, the upper cheese surface was inoculated with three spots of 50 spores of either *P. commune* or *M. racemosus* as fungal targets. For positive controls, instead of bioprotective cultures, natamycin (LCP natose, Laboratoires Humeau, France) was applied by spray over the upper surface of the cheese at a final concentration of 0.5 mg/dm^2^ before spore inoculation. Some cheeses without spore inoculation were also included for microbial, biochemical analyses, shelf life tests and sensory evaluations. All cheeses were ripened for four weeks at 12°C and 96% relative humidity (1 chamber per fungal target). Cheeses were sampled aseptically for pH measurement, microbial analyses of starters and antifungal cultures over a period of 4 weeks at 1 week intervals.

### Bacterial growth and physicochemical parameters

To evaluate the growth of antifungal strains and their potential impact on the commercial lactic starters, bacterial populations were enumerated before and after fermentation as well as during storage (once a week). To do so, sour cream (1 g) samples were diluted in sodium chloride peptone broth and semi-hard cheese (10 g) samples were blended in sterile bags with 2% trisodium citrate (Sigma-Aldrich, Saint-Louis, MO, USA) using a stomacher (Merck Eurolab, Strasbourg, France). Antifungal combinations were cultivated on LAMVAB agar (Hartemink et al., 1997) and incubated under anaerobic conditions (Anaerocult A; Merck, Darmstadt, Germany) at 30°C for 5 days. MM100 and MA016 starters were cultivated on M17 agar (Difco Laboratories, USA) supplemented with 0.5% lactose and incubated for 24 h at 30°C in aerobic conditions.

### Shelf life tests

Shelf life tests were performed to evaluate the antifungal activity exerted by the selected associations in the produced sour cream and semi-hard cheeses (free from fungal targets), in a naturally contaminated environment. For sour cream, after 2 weeks of storage at 4°C, 200 g of each condition were spread out in ten 100 mm Petri dishes. The plates were then exposed for 20 min in a lunch room (INRA, STLO), then sealed with plastic paraffin film and incubated at 10°C. Concerning semi-hard cheeses, 3 weeks ripened semi-hard cheeses of each condition were sliced (60 mm x 50 mm x 8 mm). The obtained slices were then exposed for 20 min in ten 60-mm Petri dishes. Plates were then sealed and incubated at 12°C. In both cases, fungal growth at the surface was visually evaluated after 10 days. Fungal contaminants were isolated on M2Lev agar (20 g/L malt extract, 3 g/L yeast extract and 15 g/L agar, 5 mg/l penicillin, 5 mg/l streptomycin). Isolates were then characterized at the genus level using phenotypic methods including macro- and microscopic observations (Pitt and Hocking, 2009) and for species identification, specific taxonomical markers were targeted. DNA regions of isolated yeasts and molds were amplified, sequenced and identified as detailed by Garnier et al. (2016).

### Sensory analyses

The impact of antifungal cultures on the sensorial properties of the produced sour creams and semi-hard cheeses was evaluated using a sorting task by a panel of 27–30 untrained judges. Sour cream samples were stored at 4°C for 15 days before sensory evaluation and 10 g of each sample was presented in disposable cups. Semi-hard cheeses were evaluated after 4 weeks ripening. Cheese rind was first brushed and wiped to harmonize the aspect of the samples and presented as 15 g (50 × 40 × 8 mm) slices. Samples were left for ~30 min at room temperature before being served, coded using a random 3-digit code and presented in a different, counterbalanced order for each judge. Control sour cream and cheese (with no antifungal culture) were presented in duplicate, leading to a total of 10 and 5 samples per session, respectively, for cream and cheese. Panelists were instructed to group together the samples perceived as the most similar, taking into account the characteristics they considered as important to differentiate the products. Once groups were made, panelists had to associate specific descriptors (Supplementary Table 1) to each group. Data were analyzed as recommended by Lê and Worch (2014) in the *R* free software using the *FactorMineR* package by generating a contingency table (*descfreq* function) that calculated the number of occurrences of each descriptor in the different samples (*p* < 0.2). Then, a correspondence analysis (CA) was performed from the contingency table and plotted with confidence ellipse (functions *plot.CA* and *ellipseCA*).

## Results

### Antifungal activity screening

Among the 32 screened strains (Table 1), lactobacilli showed higher antifungal activities than leuconostocs and propionibacteria. All strains except *Propionibacterium acidipropionici* CIRM-BIA928 showed antifungal activity against at least 1 fungal target in model cheese or yogurt. The antifungal activity varied between strains for all tested species. *P. commune* was the most inhibited fungal target followed by *M. racemosus*, while the *G. geotrichum* and *Y. lipolytica* yeasts were the most resistant as they were only inhibited by 1 *Leuconostoc* and 6 lactobacilli strains, with a very low (+) or slight (++) antifungal activity.

Antifungal strains showed a specific antifungal activity in each dairy model. The global score, i.e., the mean score of the 4 fungal targets tested, showed that 25 strains exhibited a higher or similar antifungal activity in cheese compared to yogurt, while it was the reverse for 6 strains (5 L. rhamnosus and 1 L. plantarum). P. acidipropionici CIRM-BIA928, Propionibacterium jensenii CIRM-BIA1774, and P. thoeni CIRM-BIA1763 were the only propionibacteria strain that exhibited antifungal activity in both models. In yogurt, 6 Propionibacterium and 1 Leuconostoc strains showed no antifungal activity. Antifungal activity for all strains was markedly higher when inoculated at 10^7^ compared to 10^5^ CFU/mL. At 10^5^ CFU/mL, all strains exhibited very low (+) or no inhibition in yogurt while 7 lactobacilli, 1 leuconostoc strains and the reference cultures X1 and X2 showed a slight (++) to total (+++) inhibition in model cheese.

From the first screening (S1), 15 LAB strains (12 lactobacilli and 3 leuconostocs noted with a ^*^ in Table 1) showed a high global score from 1.25 to 2.75 at least in one model. Among propionibacteria, the highest global score 0.75 was observed for 3 strains including P. acidipropionici CIRM-BIA1664 and CIRM-BIA1763, and P. jensenii CIRM-BIA1785. Among the strains exhibiting the highest antifungal activity, 12 were selected for the second screening. Reference cultures X1 and X2 showed high global scores ranging from 1.25 to 2, and X1 inhibited P. commune and M. racemosus 3–4 days more than X2.

The second screening (S2) showed little variations of the antifungal activity with the different commercial starters, indicating that starters did not impact the antifungal activity of the 12 strains (Table 1).

Based on the screening data (Table 1), 5 lactobacilli strains (noted with ^**^ in Table 1) were selected based on their antifungal activity equal or higher than that of the reference cultures and/or their activity spectrum in at least one dairy model. The selected strains were Lactobacillus harbinensis L172, which showed the highest global scores in both models, L. brevis L128 and L. plantarum L244, which showed the highest global scores in model cheese, and L. plantarum CIRM-BIA1113 and CIRM-BIA1112, which showed the broadest activity spectra.

### Antifungal activity of strain combinations

#### Combination test

Based on the 5 selected strains, the 10 possible binary and 3 ternary strain combinations were evaluated in the yogurt and cheese models. Ternary combinations were configured to test the effect of combining the high antifungal activity of L. harbinensis L172, L. brevis L128, and L. plantarum L244 with 1 of the 2 L. rhamnosus strains that showed antifungal activity against the resistant G. geotrichum and Y. lipolytica fungi. However, these ternary combinations did not show any antifungal activity improvement compared to the corresponding pure cultures and binary associations (data not shown). Four binary combinations, A1, A2, A3, and A4, showed the highest antifungal activity among the 13 tested combinations (data not shown). The four binary combinations involved 4 strains, in particular L. plantarum L244 used in 3 combinations, and L. harbinensis L172 and L. brevis L128, used in 2 combinations.

In yogurt, A1 and A2 combinations showed the highest inhibition time (4 days) on P. commune growth in the third screening. The inhibition obtained with the A3 and A4 combinations, 2 or 3 days for P. commune, was similar to pure cultures having the highest antifungal activity (i.e., L. plantarum L244 and L. harbinensis L172). Combination A4 showed the highest antifungal activity against G. geotrichum in yogurt with an inhibition for 1 day vs. no or low inhibition and no or slight inhibition for the other combinations.

In cheese model, combinations A1, A2, and A3 showed a broad spectrum, with a high inhibition of 1–4 days of P. commune and M. racemosus, and a slight to total inhibition of G. geotrichum and Y. lipolytica. Regarding inhibition of P. commune, the number of inhibition days induced by A1, A2, A3, and A4 were similar to that of the strains that showed the highest inhibition in pure cultures. A1 and A2 combinations showed inhibition of 1 day of G. geotrichum, while A2 was the only combination inhibiting Y. lipolytica growth for the same duration.

Before pilot scale applications, a safety evaluation of the strains composing the different combinations was performed. In this context, biogenic amine production and antibiotics resistance were studied. Combinations A2 and A4 were excluded because of the risk of biogenic amine production by L. brevis L128, which possesses both the tyrDC and agDi genes, as recently demonstrated (Coton et al., 2018). These genes, encoding for a tyrosine decarboxylase and an agmatine deiminase, are associated with potential production of tyramine and putrescine, respectively. Therefore, only the A1 and A3 combinations were then kept for following experiments.

#### Determination of the optimal inoculum for antifungal activity and action spectrum

The minimal inoculum allowing high antifungal activity was assessed using 3 intermediate levels of inoculation for combinations A1 and A3, selected from the previously obtained results.

In yogurt, the highest antifungal activity was always observed when strains were inoculated at the highest level, i.e., 2.10^7^ CFU/mL (Table 2). In contrast, in cheese, inoculum could be lower depending on the target (10^7^ CFU/mL and even 2.10^6^ CFU/mL in some cases). Combinations A1 and A3 showed little variations of antifungal activity with the different tested commercial starters (data not shown).

**Table 2:**
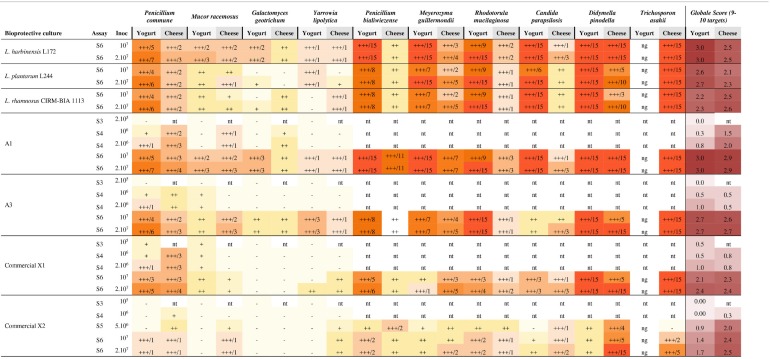
Antifungal activity of 3 selected strains and their binary combinations (A1 and A3) in yogurt and cheese model against 10 fungal targets.

*L., Lactobacillus; A1, Lactobacillus harbinensis L172 and L. plantarum L244; A3, L. plantarum L244 and L. rhamnosus CIRM-BIA1113; X1 and X2, antifungal reference cultures (for details see the Materials and Methods section)*.

*Antifungal activity obtained in the different assays: S3, Third screening of 5 selected strains in 13 combinations; S4, Fourth screening the selected combinations for determination of optimal inoculum; S5, Fifth screening of combinations against 10 fungal targets; and S6, Sixth screening of A1, A3 and their pure cultures against 10 fungal targets. Inoc,inoculum in CFU/mL*.

*Antifungal activity in the dairy products: Yogurt and Cheese with commercial starter MY800 and MA016, respectively. Nt, not tested*.

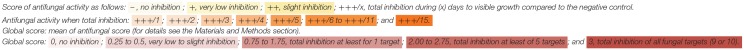

As for the antifungal activity spectrum, the six additional fungal targets (P. bialowiezense, P. pinodella, R. mucilaginosa, M. guilliermondii, C. parapsilosis, and T. asahii) were more sensitive than the initial 4 fungal targets. In particular, the time to visible growth reached at least 1 day and more than 15 days in many cases (Table 2). It should be noted that T. asahii did not grow in yogurt therefore the inhibition against this target could not be established in this model. Combination A1 showed the widest spectrum and led to the highest inhibition time in both models against the six additional fungal targets with a global score of 3 in yogurt and cheese, and was associated with 2–15 days of inhibition (Table 2). At the two levels of inoculum (10^7^ and 2.10^7^ CFU/mL) in yogurt, the A3 combination totally inhibited at least 6 of the 9 tested fungi, for a duration ranging from 2 to 8 days. The A3 combinations showed a similar spectrum than that of A1. Nevertheless, A1 did not inhibit R. mucilaginosa (yogurt), and only and slightly inhibited M. guilliermondii and R. mucilaginosa (cheese). Combination A3 showed a slight inhibition of R. mucilaginosa and C. parapsilosis in yogurt.

The activity spectrum of A1 and A3 was compared to the corresponding pure cultures, against 10 fungal targets in cheese model and 9 in yogurt, at 10^7^ and 2.10^7^ UFC/mL (Line S6 from Table 2). In half of the cases, the time to visible growth increased by 1–11 days for the combinations compared to the corresponding pure cultures. In 64% of cases, it did not differ from the strain showing the highest activity in pure culture, and in 4% of cases, it decreased.

### In situ application of antifungal combinations in sour cream and semi-hard cheese

These pilot scale applications consisted in evaluating the ability of two selected combinations, A1 and A3, to inhibit fungi in challenge and shelf life tests in sour cream and semi-hard cheese.

#### Challenge-tests

Inoculation of the fungal targets was performed to simulate a post-processing contamination. Potassium sorbate and natamycin were used as chemical additive reference (positive controls) for sour cream and semi-hard cheese, respectively.

**Figure 2:**
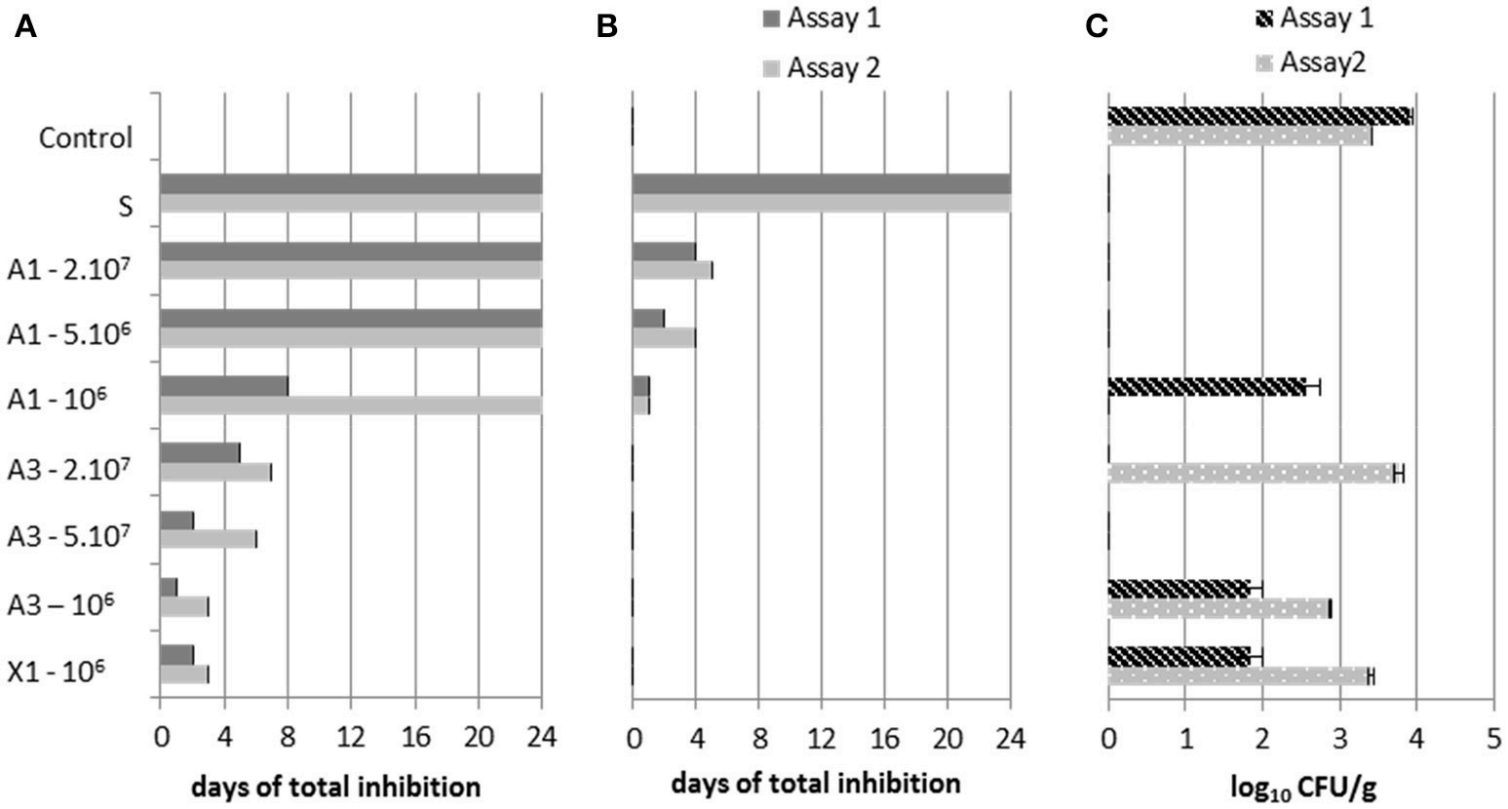
Effect of combinations A1, A3 and X1 (for details see the Materials and Methods section) at different inoculation levels 2.10^7^, 5.10^6^, and 10^6^ CFU/mL, sorbate (S, positive control) and control (negative control, no antifungal agent added) in sour cream against P. commune **(A)** and M. racemosus **(B)** (antifungal activity was expressed in days to visible growth compared to the negative control); and R. mucilaginosa **(C)** (yeast counts after storage for 7 days at 10°C, expressed as log_10_ CFU/g).

In sour cream, the fungal target P. commune was the most inhibited, followed by R. mucilaginosa and M. racemosus, which were inhibited during a maximal time of 7 days compared to 24 days for P. commune (Figure 2). The A1 combination showed higher inhibition of the 3 targets compared to A3 and the reference culture X1, and an equal inhibition to that of sorbate (24 days) against P. commune, although some variability was observed. A1 antifungal activity led to 1 to 24 days inhibition of M. racemosus and P. commune (Figures 2A,B). In sour cream containing A1 at the 3 inoculum levels, R. mucilaginosa was not detectable (<1 cell/g) after 7 days (Figure 2C). At the 3 tested concentrations, A3 slightly inhibited (++) M. racemosus (data not shown) and totally inhibited P. commune for 1–7 days (Figure 2A). In one of sour cream assays, A3 only inhibited R. mucilaginosa at a 5.10^6^ CFU/mL inoculum level (Figure 2C). X1 inoculated at 10^6^ CFU/mL showed a similar antifungal activity to that of A3 inoculated at the same concentration. Some variability in antifungal activity was observed between replicates. This was especially true for inhibition of P. commune and R. mucilaginosa by the A1 combination at the lowest inoculation level.

**Figure 3:**
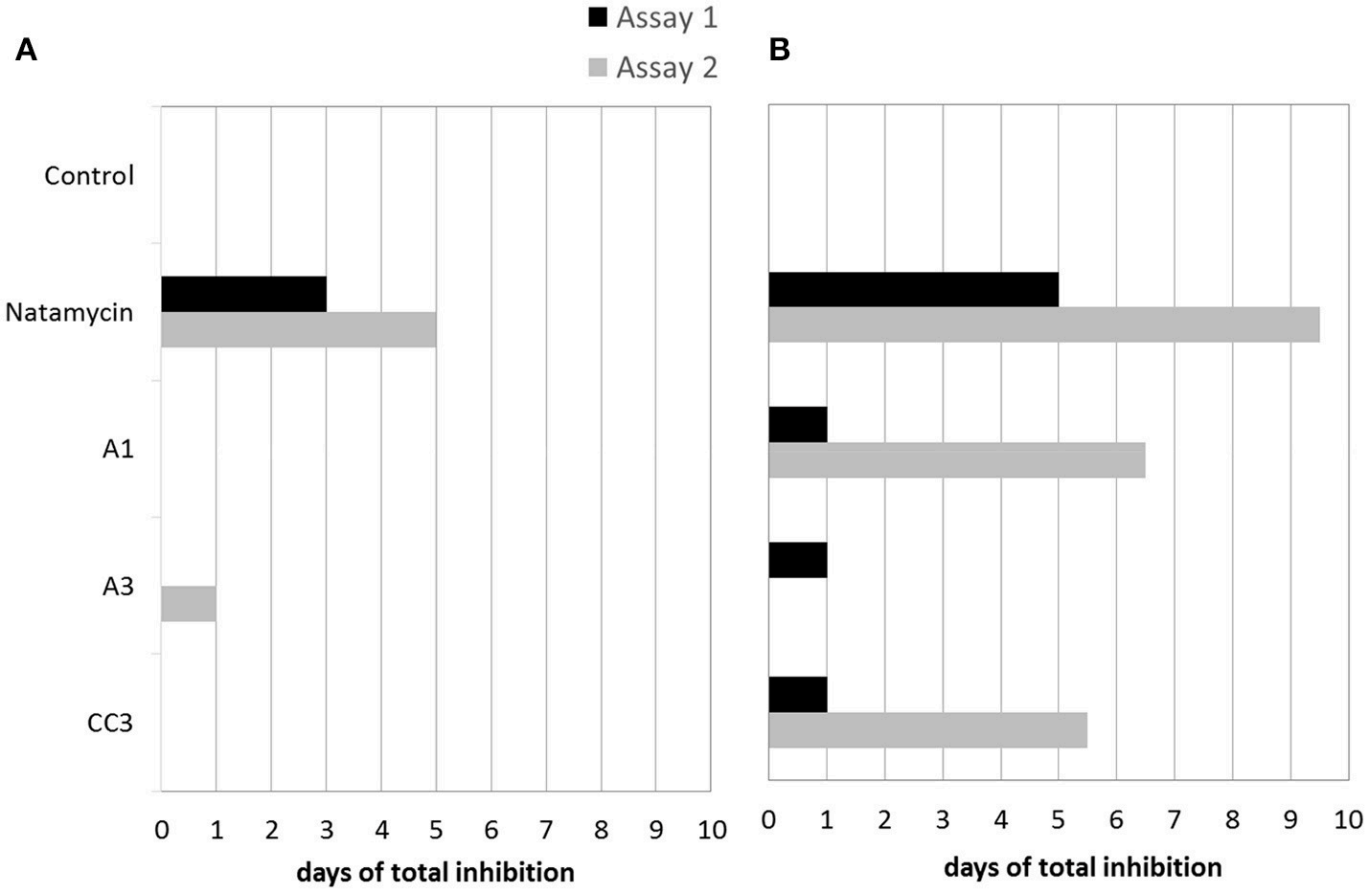
Antifungal activity of natamycin, and the combinations A1, A3 and commercial antifungal culture CC3 inoculated at 5.10^6^ CFU/mL on semi-hard cheese against P. commune **(A)** and M. racemosus **(B)** (antifungal activity was expressed in days to visible growth compared to the negative control).

In semi-hard cheeses, in the second assay, M. racemosus was totally inhibited for 1 day by A3 (Figure 3) and slightly inhibited (++) by A1 (data not shown). The fungal target P. commune was more inhibited by A1 and the commercial culture CC3, 1 to 6 days depending on the assay (Figure 3A). Antifungal activity of bioprotective cultures and natamycin varied between assays.

#### Shelf life tests

**Figure 4:**
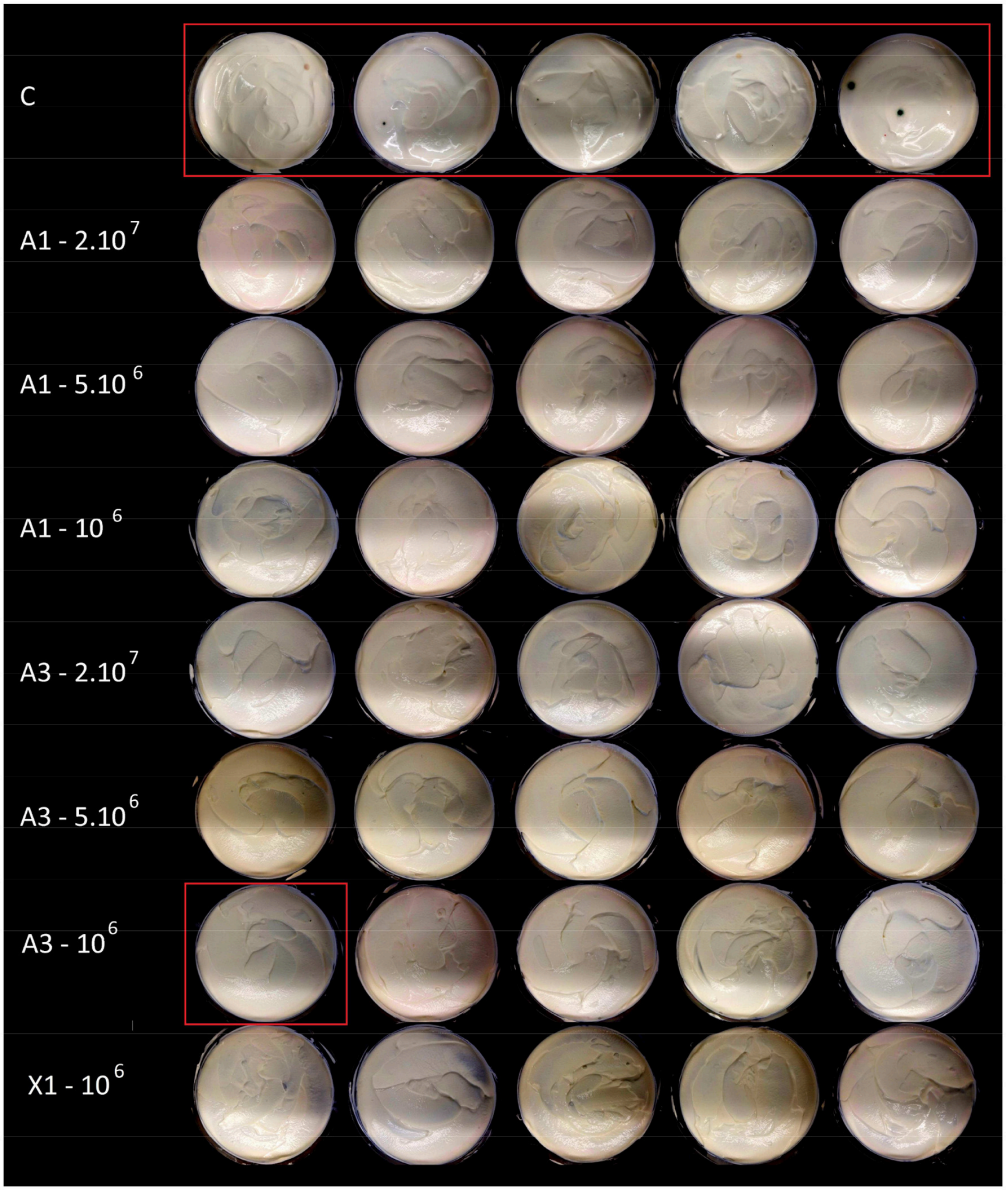
Photographs showing sour cream surfaces exposed to natural contamination by environmental fungi followed by incubation for 10 days at 10°C. Cream with no antifungal agent (C), and antifungal cultures A1, A3, and X1 (X1 = reference culture, for details see the Materials and Methods section). Inoculation levels are expressed in CFU/mL and contaminated samples are highlighted in red.

**Figure 5:**
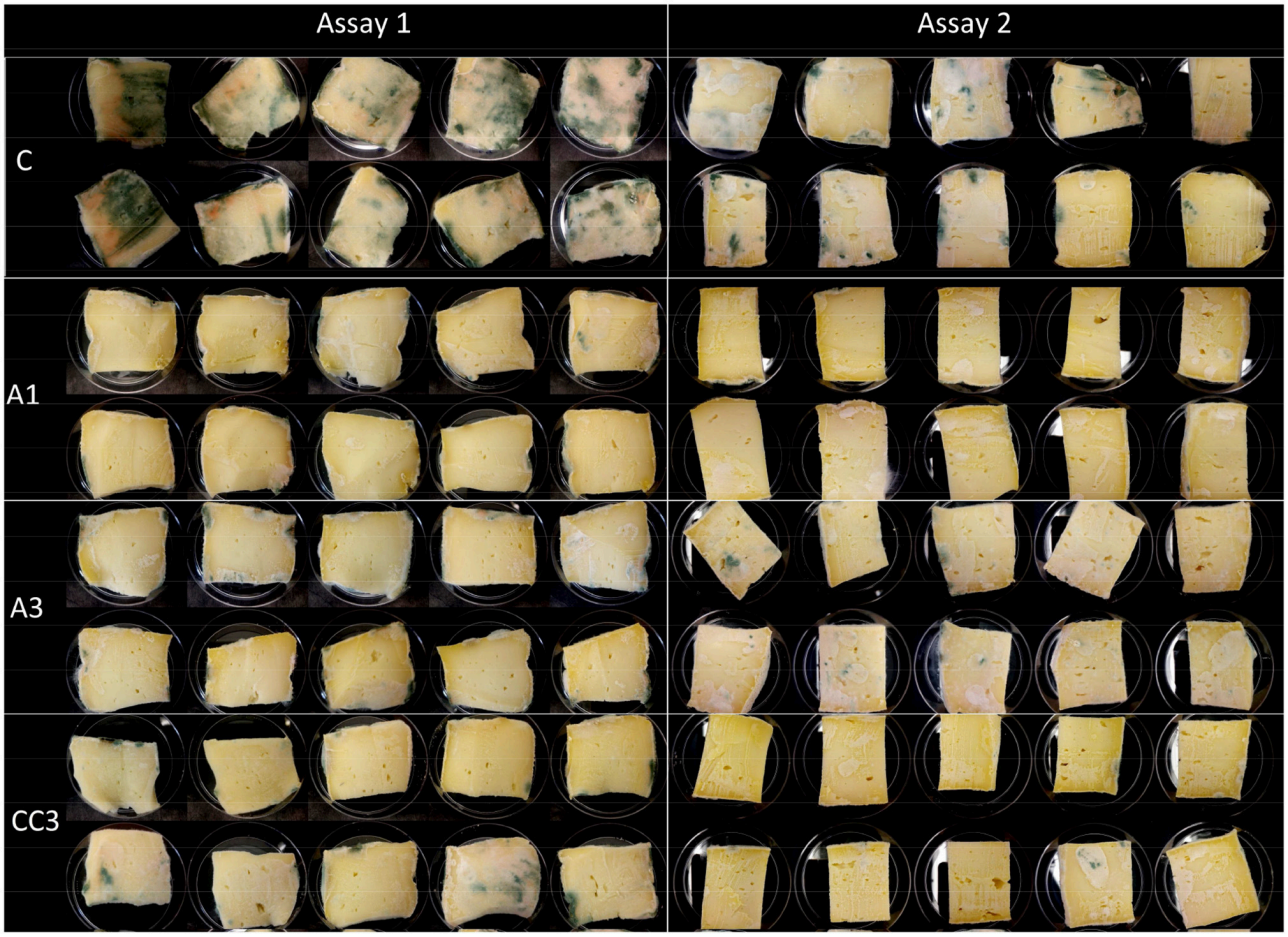
Photographs showing semi-hard cheese slices exposed to natural contamination by environmental fungi followed by incubation for 10 days at 10°C. Cheeses with no antifungal agent (Control) and antifungal cultures A1, A3, and CC3 (commercial antifungal culture) at 5.10^6^ CFU/mL.

Shelf life tests were performed on sour cream and semi-hard cheese to evaluate the growth of naturally contaminating fungi and to get closer to actual food post-contamination in particular in terms of inoculum (~1 spore in natural contamination vs. 50 spores as used as a worst case scenario in the previous experiments). For negative control sour creams (without potassium sorbate or antifungal culture), 100% of samples were contaminated. In contrast, for all the other treatments, no contamination of cream samples was observed with exception of the sour cream inoculated with A3 at 5.10^6^ CFU/mL, which presented 10% of contaminated samples (Figure 4). The natural fungal contaminants found on contaminated sour cream with A3 were identified as Cladosporium allicinum and Exophiala xenobiotica, while the fungal contaminants found in control sour cream included the 2 later mentioned fungi and 6 other species, namely Penicillium crustosum, Penicillium glabrum, Cladosporium cladosporioides, D. hansenii, Bulleromyces albus, and Sporidiobolus metaroseus. In semi-hard cheese, it is worth noting that after 4 weeks in non-sterile ripening chambers at 12°C, the cheese surface was colonized in all assays with molds, therefore fungal growth was evaluated on the slice surface after 10 days of incubation post-slicing. The totality of negative control cheeses samples were contaminated, whereas samples of A1, A3 and commercial culture CC3 supplemented cheeses were notably less contaminated as shown on Figure 5. The fungal contaminant on cheese slices was identified as Penicillium crustosum.

#### Microbial enumeration and pH

Antifungal populations were evaluated by enumeration of the total lactobacilli in sour cream and semi-hard cheese in order to verify their viability at inoculation time, their growth and survival at different stages of manufacture and storage. Samples containing A1 and A3 presented two types of lactobacilli colonies with similar number on the plates, corresponding to the two inoculated strains constituting the antifungal combinations.

**Figure 6:**
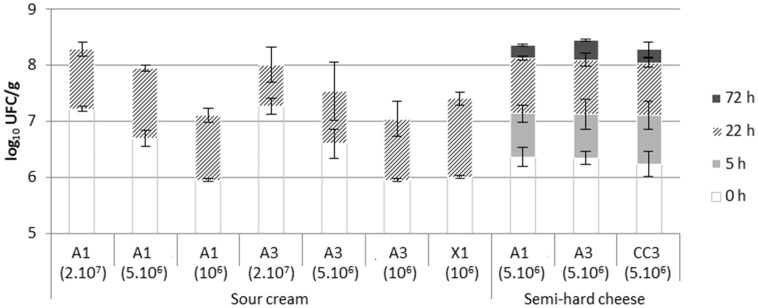
Populations of antifungal bacteria (A1, A3, X1, or CC3, see Materials and Methods section for details) inoculated at 2.10^7^, 5.10^6^, and 10^6^ CFU/mL in sour cream and semi-hard cheese at inoculation time (0 h) and at the end of fermentation (22 h) and, for cheese only, after curd press (5 h) and smear treatment (72 h).

The determined populations of antifungal cultures in cream and cheese milk were in agreement with the targeted inoculum concentrations and showed similar changes during the process regardless of the considered antifungal culture. A1 and A3 combination populations were stable or even increased during cheese ripening with maximal values of ~8.5 log_10_ CFU/g (Figure 6).

There was no significant difference of pH during acidification between sour cream with and without antifungal cultures. Nevertheless, after 4 weeks of storage at 10°C, the pH of cream inoculated at 2.10^7^ CFU/mL with both A1 and A3 combinations was lower (0.2 pH units less) than that of control creams. In semi-hard cheese, antifungal cultures did not impact pH neither during manufacture nor during ripening.

Starter culture populations in sour cream and semi-hard cheeses with antifungal cultures did not differ, during the fermentation and 4 weeks of storage/ripening, from those of the control samples (data not shown). No NSLAB (Non Starter Lactic Acid Bacteria) was detected on LAMVAB on the control products. Bacterial population slightly varied during storage/ripening or sour cream and semi-hard cheese. In all products, starter culture population decreased in both products from the second weeks of storage. Concerning antifungal cultures, A1, A3 and X1 population increased during the first week of storage in sour cream. In semi-hard cheese, A1 and A3 population slightly increased during the 4 ripening weeks (data not shown).

#### Sensory analyses

**Figure 7:**
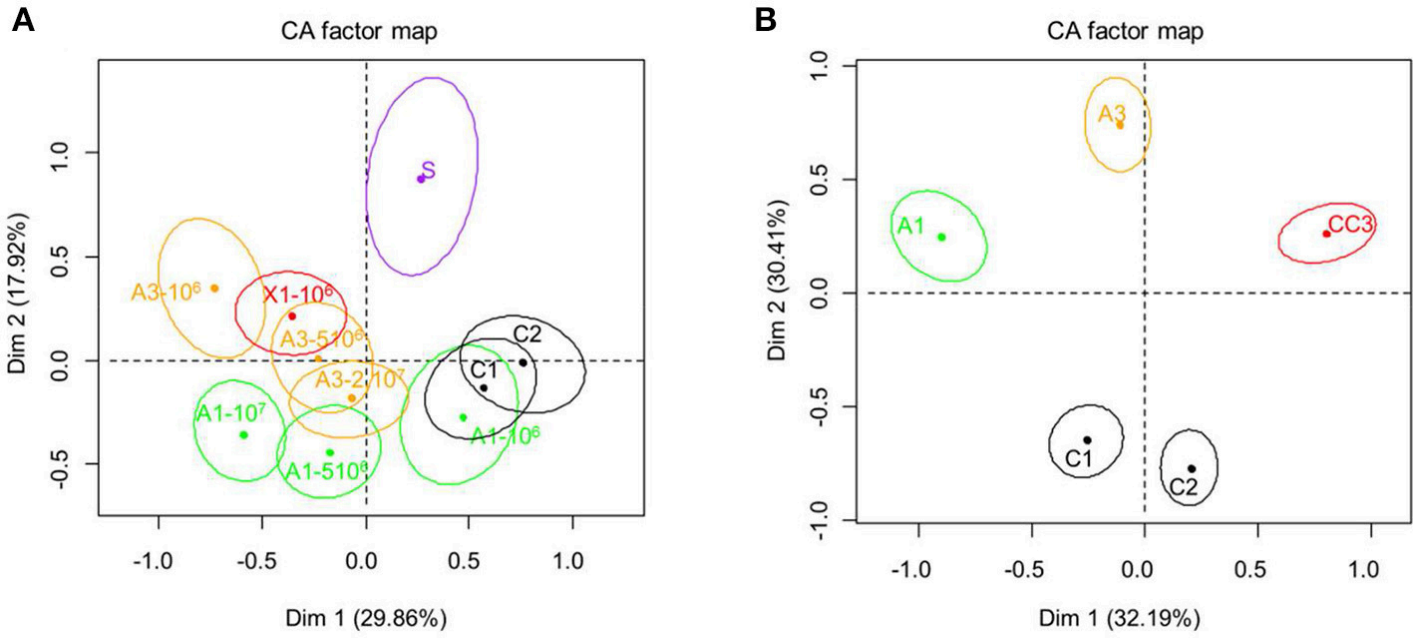
Correspondence analysis map of sensory analysis data obtained for the sour cream **(A)** and semi-hard cheeses **(B)** samples. For sour cream samples: no antifungal agent (negative controls = C1 and C2), potassium sorbate at 0.08% (S) (positive control) and antifungal cultures A1, A3, and X1 (see Materials and Methods section for details) at 2.10^7^, 5.10^6^, and 10^6^ CFU/mL. For semi-hard cheeses: no antifungal agent (negative controls = C1 and C2), antifungal bioprotective cultures A1, A3, and CC3 (commercial antifungal culture) at 5.10^6^ CFU/mL.

The CA map built from sensory evaluation data of sour cream showed that the samples were mainly separated on dimension 1 and cream with sorbate was separated from all other samples on dimension 2 (Figure 7A). The negative side of dimension 1 in the map contained 6 out of the 7 creams with antifungal cultures including the reference culture X1, characterized by their more acidic flavor (Table 3). Only the cream with A1 at 10^6^ CFU/mL was perceived similar to the control samples and characterized by a “mild flavor.”

**Table 3:** Descriptors cited at a significantly (p < 0.2) higher frequency between sour creams (assay 1).

**Sample**	**Inoc**.	**Descriptors**	**Intern %**	**p-value**
Control 1	–	Tasteless	7.8	0.04
		Odorless	6.3	0.06
		Mild flavor	12.5	0.11
Control 2	–	Mild flavor	13.4	0.05
A1	2.10^7^	Acidic	23.4	0.03
		After-taste	3.1	0.16
	5.10^6^	Lactic	6.2	0.14
	10^6^	Lactic	15.2	0.02
		Nutty after-taste	3.0	0.17
A3	2.10^7^	Slightly acidic	6.0	0.18
	5.10^6^	Balanced	3.2	0.10
	10^6^	Very acidic	11.5	0.04
		Pungent	11.5	0.09
		Acidic	21.3	0.10
		Pronounced flavor	1.6	0.19
X1	10^6^	Mild flavor	3.3	0.15
		Sour cream	1.6	0.19
		Acidic	19.7	0.20
S	–	Sweet	3.1	0.02
		Different after-taste	3.1	0.10
		Different taste	3.1	0.16
		Cheese flavor	1.6	0.20

*Control 1 and 2, same sample, sour creams with no antifungal culture. A1, A3, and X1, sour cream inoculated with the antifungal cultures A1, A3, and X1 (X1 = reference culture, for details see the Materials and Methods section), S, Sour cream with no antifungal culture and 0.08% of potassium sorbate, Inoc., Inoculum of the antifungal culture (CFU/mL), Intern %, Percent of frequency for each descriptor inside the group. Descriptors were translated from french to english, for more details go to Supplementary Table 1*.

For cheeses, the CA map separates control cheeses from the other cheese samples on dimension 2 and the cheese containing the A1 culture on dimension 2. Control cheeses were characterized as tasteless, whereas cheese with CC3 was described as slightly acidic and with a different texture, and cheese A1 associated with a more bitter and acidic flavor (Figure 7B, Table 4).

**Table 4:** Descriptors cited at a significantly (*p* < 0.2) higher frequency between semi-hard cheese inoculated with antifungal combinations A1, A3 and commercial antifungal culture CC3 at 5.10^6^ CFU/mL and the non-inoculated control semi-hard cheeses (Control 1 and 2).

**Sample**	**Descriptors**	**Intern %**	***p*-value**
Control 1	Tasteless	14.05	0.07
Control 2	Tasteless	16.36	0.01
	Not melting	1.82	0.16
A1	Some holes	1.69	0.07
	Bitter taste	1.69	0.07
	Pungent	5.08	0.09
	Odorless	2.54	0.10
	Acidic	4.24	0.15
A3	No significantly different descriptors		
CC3	Smooth texture	1.89	0.03
	Slightly acidic	4.40	0.05
	No holes	1.26	0.13
	Melting	1.26	0.13
	Firm texture	2.52	0.14

*Control 1 and 2, same semi-hard cheese sample with no antifungal culture. A1, A3, and CC3, semi-hard cheese inoculated with antifungal cultures A1, A3, and CC3 (CC3, commercial antifungal culture). Intern%, Percent of frequency for each descriptor inside the group. Descriptors were translated from french to english, for more details go to Supplementary Table 1*.

## Discussion

In the first part of this work, the antifungal activity of 32 strains for potential use as adjunct antifungal cultures was evaluated using a high-throughput screening method in two dairy models against 4 fungal targets, namely *P. commune, M. racemosus, G. geotrichum*, and *Y. lipolytica*. As shown in several studies, the choice of the screening media is a crucial step because the number of potential antifungal agents can be drastically reduced between a screening step performed in synthetic media and *in situ* tests in actual food products. For example, Delavenne et al. (2013) indicated that from 11 antifungal LAB active *in vitro*, only 1 showed a very strong antifungal activity in yogurt. Similarly, Le Lay et al. (2016b) showed that the number of LAB active in bakery products was significantly lower than that observed on MRS and WFH media. In order to avoid this bottleneck, in this study, two media closely related to actual dairy products were chosen, a cheese-mimicking model previously developed by Garnier et al. (2018) and yogurt adapted to high-throughput screening in this study. They were both relevant to the final purpose of developing antifungal cultures compatible with various dairy technologies. The use of this screening method allowed preparing more than 5,000 miniaturized yogurts and model cheeses required to test the antifungal candidates, in the presence of different commercial starters, against up to 10 fungal targets. A difference of antifungal activity was observed between the two dairy models, thus demonstrating the importance of matrix (composition) and production condition (time of incubation, temperature). No significant impact of the 5 commercial starters tested in yogurt and cheese models was observed on the antifungal activity. This suggested low interaction (metabiosis) between the starter cultures and the antifungal adjunct cultures. The choice of fungal targets is also crucial as they should be representative of contaminants in terms of occurrence and biodiversity. In this study, fungal target strains were selected from contaminated dairy products (Garnier et al., 2016) among species known as frequent contaminants in dairy products (Pitt and Hocking, 2009). Noteworthy, in the present study, the 4 fungal targets used for the screening steps were more resistant, in particular *G. geotrichum* and *Y. lipolytica*, than the 6 additional fungi used during the activity spectrum evaluation. In agreement with our observations, the two latter species were reported to be more resistant to chemical preservatives than other fungal species, e.g., for *G. geotrichum* and *Y. lipolytica* strains, the minimal inhibitory concentration of potassium sorbate at pH 5 in PDA was 1 g/L in contrast to 0.3 g/L for *P. commune* (Garnier et al., 2016). Similarly, the antifungal activity of LAB strains against cereal and bakery fungal contaminants was reported to differ for all the tested fungal targets (Inglin et al., 2015; Le Lay et al., 2016b; Russo et al., 2017).

The tested *Lactobacillus* strains globally showed the highest antifungal activity in one or both of the dairy models, compared to *Leuconostoc* and *Propionibacterium* strains, even if the activity was strain-dependent within the species, as expected. Many *Lactobacillus* species have been previously reported to exhibit antifungal activity in dairy products. For example, in yogurt, several authors showed that various strains belonging to the *L. harbinensis, L. rhamnosus, L. paracasei* or *L. casei* species were active against *Penicillium* spp., but also, for some of them, against *D. hansenii, K. lactis, Kluyveromyces marxianus, R. mucilaginosa*, and *Y. lipolytica* (Delavenne et al., 2013, 2015; Li et al., 2013; Aunsbjerg et al., 2015). In cottage and cheddar cheese, different studies showed that *L. reuteri, L. amylovorus, L. plantarum* and *L. rhamnosus* strains showed antifungal activity against *Penicillium* spp. (Cheong et al., 2014; Lynch et al., 2014; Fernandez et al., 2017). Assessment of the antifungal activity of 13 LAB strains toward 3 ochratoxin A-producing fungi in synthetic media showed that *L. plantarum* B4496*, L. brevis* 207 and 3 other LAB strains inhibited the tested fungal targets (Ngang et al., 2015). The lower antifungal activities observed for *Leuconostoc* and *Propionibacterium* strains, despite their reported capacity to produce antifungal organic acids, could result from their inability to reach high populations under the screening conditions (i.e., incubation for 4–6 h at 42°C in yogurt and for 72 h at 20°C in cheese). *Leuconostoc* spp. optimally grow at temperatures ranging between 20 and 30°C (Martley and Crow, 1993; Hemme and Foucaud-Scheunemann, 2004). This aspect may explain the low antifungal activity of *Leuconostoc* strains observed in the present study in yogurt. Concerning *Propionibacterium* spp., their slow growth rate can explain their low activity observed. The generation time of *P. freudenreichii* is around 5–9 h under optimal conditions, i.e., in a complex laboratory medium at 30°C (Thierry et al., 2002), and 15–30 h in cheese and cheese juice at 24°C (Salvat-Brunaud et al., 1997). Antibiosis, via the production of antifungal molecules, and pH decrease are the main factors contributing to LAB and PAB antifungal activity in foods (Leyva Salas et al., 2017). The production of antifungal compounds (e.g., lactic, acetic, succinic, propionic, formic, and butyric acids) by lactobacilli and pediococci strains was shown to vary significantly depending on the medium (Özcelik et al., 2016). There are only few studies concerning the antifungal activity of *Leuconostoc* compared to those concerning *Lactobacillus* spp. and most of them were performed *in vitro*. For example, two *L. citreum* strains were shown to inhibit fungal contaminants of bakery products (Valerio et al., 2009; Baek et al., 2012; Le Lay et al., 2016b).

The binary combinations A1 and A3 showed the same broad spectrum as their constituting strains grown in pure cultures. They increased the time to visible growth of fungal targets in one third of cases (inhibition tested for 2 combinations, at 2 inoculations levels against 10 fungal targets in two dairy models). Some previous studies performed in different media also reported an improvement of the antifungal activity of binary combinations of strains, compared to pure cultures. This was the case for example for combinations of *L. paracasei* subsp. *paracasei* SM20 and *P. jensenii* SM11 *in vitro* (Schwenninger and Meile, 2004), *P. thoenii* P-127 and either *L. rhamnosus* B-445 or *L. plantarum* DSA 20174 in Kareish cheese (El-Shafei et al., 2008), and *L. rhamnosus* A238 and *Bifidobacterium animalis* subsp. *lactis* A026 in cottage cheese (Fernandez et al., 2017). The WO2012136830A1 patent applicable to yogurt reported that binary combinations (*L. paracasei* CHCC12777 and *L. rhamnosus* CHCC12697 or *L. paracasei* CHCC12777 and *L. rhamnosus* CHCC14226) were more effective than either of the strains alone (Hornbaek et al., 2014). We hypothesized that the improvement of the antifungal activity of binary combinations consisting of *L. plantarum* L244 combined with either *L. harbinensis* L172 (A1), or *L. rhamnosus* CIRM-BIA1113 (A3) observed in this study was due to a higher quantity and diversity of the antifungal compounds produced compared to single strains, because it is known that antifungal compounds act in synergism and that *Lactobacillus* spp. produce various antifungal compounds (Crowley et al., 2013; Aunsbjerg et al., 2015; Le Lay et al., 2016a). Despite the fact that the A1 and A3 combinations most generally did not improve the antifungal activity to a great extent compared to the corresponding strains, we expect that using a combination of strains rather than a single strain would increase the technological flexibility and the chance to see at least one of the strain expressing its antifungal activity in a diversity of conditions.

The *in vitro* screening and the challenge-tests performed in the present study are a worst case contamination scenario because 50 fungal spores were inoculated due to practical constraints, far above a natural contamination generally due to 1–2 contaminant spores. Moreover, cream was incubated at 10°C instead of 4°C as usually recommended for storage. Taken together, these aspects suggest that the antifungal activity could be even higher in real conditions, as strongly suggested by the shelf life test results. In sour cream, A1 and A3 combinations showed a bioprotective activity against natural fungal contaminants at all tested inoculum levels even the lowest (10^6^ CFU/mL). This demonstrated the robustness of the strategy applied in this work. There are only few reports comparing the antifungal activity of bioprotective cultures in both challenge-test and natural contamination in real products (shelf life test). A study on the antifungal activity of 2 *L. amylovorus* strains used as adjunct cultures in miniature Cheddar cheese showed that the 10^3^ spores inoculated of *P. expansum* were inhibited for 1–4 days, while the airborne contaminants were inhibited for 6 days (Lynch et al., 2014). Some studies showed the effectiveness of LAB *in situ* against natural contaminants, e.g., *L. amylovarus* DSM1928 active in wheat, gluten-free and quinoa bread (Axel et al., 2015, 2016a,b), a semi-liquid bio-preserver (SL778) developed from *L. plantarum* CRL 778 in bread (Gerez et al., 2015), and fermentates of *P. jensenii* and *L. rhamnosus* in sour cream and semi-hard cheeses (Ganier et al. submitted).

Some of the constraints related to the potential use of bioprotective cultures (Leyva Salas et al., 2017) were also considered in this study, including safety, sensory and economic constraints (level of inoculation). From *in vitro* tests in dairy products to the scale up in sour cream and semi-hard cheese work, we designed the study to reduce the gap often observed between the antifungal activity *in vitro* and *in situ*. Concerning the safety of the strains to be used as adjunct cultures, although lactobacilli are not known as pathogens, it is necessary to follow a rigorous safety assessment procedure, taking multiple criteria into account. Recently, biogenic production and antibiotic resistance pattern was studied in some of the strains used in this study (Coton et al., 2018). In the latter study, *L. brevis* L128 was identified as a biogenic amine-producer, which precludes its use as a potential bioprotective culture, and led us to exclude the A2 and A4 combinations including this strain, despite its high antifungal activity. Beyond the latter activity, it is crucial to evaluate the impact of the adjunct cultures on the sensory properties of the treated dairy products. The sensory proximity of the creams with the reference culture X1 and the creams with A1 and A3 suggests that the selected combinations could also be used without a marked adverse impact on cream sensory properties. Concerning the more acid flavor reported by the judges in the creams containing A1 and A3, it could be linked to a post-acidification increase compared to the products without adjuncts. Similarly, some commercial antifungal cultures used as antifungal in dairy products are known to increase post-acidification, especially when the products are stored at ambient temperatures (Nielsen et al., 2017). Despite organoleptic differences and post-acidification at high inoculum levels, the dairy products containing the antifungal combinations were still acceptable by the judges.

The minimal efficient inoculum concentration is important from an economic point of view. An initial concentration of 10^7^ CFU/mL (i.e., 5.10^6^ CFU/mL of each strain) was efficient in both models from this work. In another study in yogurt, it was demonstrated that an inoculum level of 10^5^ CFU/mL of *L. harbinensis* K.V9.3.1Np was necessary to get an antifungal activity, but 5.10^6^ CFU/mL was required for a total inhibition (Delavenne et al., 2015). To explain the variation in the antifungal activity of A1 observed in sour cream at the lowest inoculation level, 10^6^ CFU/mL, we can hypothesize that a minimal limit of population at the moment of contamination is required to express an inhibitory effect. A similar phenomena was reported in yogurt as to inhibit *Y. lipolytica* by *L. harbinensis* K.V9.3.1Np, 2.5–3.10^6^ CFU/g of *L. harbinensis* were necessary at the moment of contamination (Delavenne et al., 2015). The authors suggested an all-or-nothing mechanism (no inhibition below organic acid MICs and total inhibition above), and a quorum sensing regulatory mechanism as in bacteriocin-production by LAB. The fact that, for a same antifungal culture, a variable antifungal activity occurred between semi-hard cheese assays also suggests a role of biotic and abiotic factors resulting from interactions between the food, the antifungal cultures and the fungal contaminants (Leyva Salas et al., 2017). The potential influencing factors include the dry matter content in cheeses, ripening chamber humidity, interactions between the smear and the fungi, and non-sterility of the ripening chambers.

This study presents an effective approach from *in vitro* screening to *in situ* application to develop antifungal combinations with high antifungal activity and broad spectrum, by covering different aspects that limit the entrance of bioprotective cultures to the market. The two selected binary combinations (i.e., A1 and A3) are good candidates for the antifungal biopreservation of dairy products.

## Author contributions

EC, AT, JM, FV, and MLS designed the experiments. MLS and ML performed *in vitro* experiments. GG, MH-O, MC, and MLS performed scale pilot experiments. SL contributed to the conception and data analysis of the sensory evaluations. MLS, AT, and EC wrote the manuscript. EC, AT, JM, and FV supervised the project. All authors discussed the results and contributed to the final manuscript.

### Conflict of interest statement

The authors declare that the research was conducted in the absence of any commercial or financial relationships that could be construed as a potential conflict of interest. The reviewer GT and handling Editor declared their shared affiliation at the time of the review.
