# Supplementary material for: Antifungal Activity of Lactic Acid Bacteria Combinations in Dairy Mimicking Models and Their Potential as Bioprotective Cultures in Pilot Scale Applications
Source: Front Microbiol. 2018 Aug 7;9:1787. doi: 10.3389/fmicb.2018.01787 (PMC6090892; doi:10.3389/fmicb.2018.01787)
Supplement: Supplementary file 1 [file Table_1.docx]

Supplementary Material

**Antifungal Activity of Lactic Acid Bacteria Combinations in Dairy Mimicking Models and their Potential as Bioprotective Cultures in Pilot Scale Applications**

Marcia Leyva Salas^1,2^, Anne Thierry^2^, Mathilde Lemaître^1^, Gilles Garric^2^, Marielle Harel-Oger^2^, Manon Chatel^2^, Sebastien Lê^3^, Jérôme Mounier^1^, Florence Valence^2^ and Emmanuel Coton^1^*

^1^Université de Brest, EA 3882 Laboratoire Universitaire de Biodiversité et Ecologie Microbienne, ESIAB, Technopôle Brest-Iroise, 29280 Plouzané, France

^2^UMR1253 Science et Technologie du Lait et de l’Œuf, INRA, Agrocampus Ouest, Rennes, France

^3^Applied Mathematics Department, Agrocampus Ouest, Rennes, France

*Correspondance:

Pr. Emmanuel Coton

[emmanuel.coton@univ-brest.fr](mailto:emmanuel.coton@univ-brest.fr)

**Supplementary 1. Descriptors used by the jury during the sensory evaluation**

| **Translation** | **Original descriptors (French)** |
| --- | --- |
| acidic | acide |
| after-taste | arrière-goût |
| balanced | équilibré |
| bitter taste | arrière-goût amer, amer |
| cheese flavor | goût fromage |
| different after-taste | arrière-goût diffèrent |
| different flavor | goût diffèrent |
| firm texture | ferme, texture compacte |
| melting | fondant en bouche |
| mild flavor | goût doux, goût faible |
| lactic | lactique |
| no holes | pas d’ouvertures |
| not melting | manque de fondant |
| nutty after-taste | arrière-goût noisette |
| odorless | odeur neutre |
| pronounced flavor | goût marqué, goût prononcé |
| pungent | piquant |
| slightly acidic | légèrement acide |
| smooth texture | texture lisse |
| a few holes | peu d’ouvertures, peu de trous |
| sour cream flavor | type crème fraîche |
| sweet | sucré |
| tasteless | fade, goût neutre, peu de goût, peu gouté, goût neutre, pas très goûtu |
